# Optimized Feature Subset Selection Using Genetic Algorithm for Preterm Labor Prediction Based on Electrohysterography

**DOI:** 10.3390/s21103350

**Published:** 2021-05-12

**Authors:** Félix Nieto-del-Amor, Gema Prats-Boluda, Jose Luis Martinez-De-Juan, Alba Diaz-Martinez, Rogelio Monfort-Ortiz, Vicente Jose Diago-Almela, Yiyao Ye-Lin

**Affiliations:** 1 Centro de Investigación e Innovación en Bioingeniería (CI2B), Universitat Politècnica de València (UPV), Camino de la Vera s/n Ed. 8B, 46022 Valencia, Spain; feniede@ci2b.upv.es (F.N.-d.-A.); gprats@ci2b.upv.es (G.P.-B.); jlmartinez@ci2b.upv.es (J.L.M.-D.-J.); adiaz@ci2b.upv.es (A.D.-M.); 2 Servicio de Obstetricia, H.U.P. La Fe, 46026 Valencia, Spain; monfort_isaort@gva.es (R.M.-O.); diago_vicalm@gva.es (V.J.D.-A.)

**Keywords:** preterm labor, electrohysterography, myoelectric activity, genetic algorithm, ensemble learning

## Abstract

Electrohysterography (EHG) has emerged as an alternative technique to predict preterm labor, which still remains a challenge for the scientific-technical community. Based on EHG parameters, complex classification algorithms involving non-linear transformation of the input features, which clinicians found difficult to interpret, were generally used to predict preterm labor. We proposed to use genetic algorithm to identify the optimum feature subset to predict preterm labor using simple classification algorithms. A total of 203 parameters from 326 multichannel EHG recordings and obstetric data were used as input features. We designed and validated 3 base classifiers based on k-nearest neighbors, linear discriminant analysis and logistic regression, achieving F1-score of 84.63 ± 2.76%, 89.34 ± 3.5% and 86.87 ± 4.53%, respectively, for incoming new data. The results reveal that temporal, spectral and non-linear EHG parameters computed in different bandwidths from multichannel recordings provide complementary information on preterm labor prediction. We also developed an ensemble classifier that not only outperformed base classifiers but also reduced their variability, achieving an F1-score of 92.04 ± 2.97%, which is comparable with those obtained using complex classifiers. Our results suggest the feasibility of developing a preterm labor prediction system with high generalization capacity using simple easy-to-interpret classification algorithms to assist in transferring the EHG technique to clinical practice.

## 1. Introduction

Premature delivery is defined as one that occurs before 37 weeks of gestation. Over 9–12% of children are born prematurely every year, this being the leading cause of new-born deaths and the second-leading cause of death after pneumonia in children under the age of 5 [1]. In the case of survivors, it is associated with 20% mental retardation, 50% cerebral palsy and 33% eye injuries [2]. Preterm births are also associated with long-term morbidity consequences such as learning disabilities, attention deficit disorder, emotional problems, respiratory distress and intraventricular hemorrhage [1]. The costs derived from premature pregnancy are significant for national healthcare systems. In the United States, the economic cost in 2005 (combined medical, educational and lost productivity) associated with preterm birth amounted to at least $26.2 billion [1]. The average first-year medical costs, including both inpatient and outpatient care, were about 10 times greater for preterm ($32,325) than for term infants ($3325) [1].

Obstetricians usually assess uterine dynamics by tocodynamometer and cervix status using cervical length and bishop scores to determine the risk of preterm labor [3]. Biochemical markers such as fetal fibronectine and interleukin-6 have also been shown to be useful to identify patients that are not at risk of preterm labor, thus obtaining a high negative predictive value [4,5]. However, all these techniques fail to detect women who will deliver prematurely, with positive predictive values lower than 0.50. Electrohysterography (EHG), which consists of the recording of uterine myoelectrical activity on the abdominal surface, has emerged as a powerful tool to predict preterm labor due to its high sensitivity in identifying the real preterm labor patients [6]. In addition to identifying uterine contractions, which is the only useful information that can be derived from tocography, relevant information on the uterine electrophysiological state can also be obtained from the EHG. Temporal, spectral and non-linear parameters are used to characterize the electrophysiological changes throughout pregnancy [7]. In this context, the EHG signal amplitude associated with the number of uterine cells involved in one contraction has been shown to increase as pregnancy progresses. As labor approaches, a shift of spectral content towards higher frequencies has also been reported, suggesting increased cell excitability [6]. Different entropy measurements such as sample entropy (SampEn), fuzzy entropy (FuzEn) and spectral entropy (SpEn) have shown that signal predictability increases as labor approaches [8], although some controversial results have been reported [7]. Likewise, signal complexity seems to decrease, which was shown by analyzing the Lempel-Ziv evolution in function of time-to-delivery [9]. Poincaré plot-derived parameters [10] have also been proposed to characterize the EHG signal, and it has been observed that signal randomness decreases as labor approaches [6].

Many efforts have focused on developing prediction models for forecasting preterm labor based on EHG features and achieved classifier accuracy of more than 95% [7]. Despite the promising results of these prediction systems, they have had no significant impact on clinical practice [11]. This is due to various factors. Firstly, most of these systems use neural networks or support vector machine, multilayer perceptron, or similar algorithms, which involved non-linear transformations of the input EHG features into high dimension space, in which data from the target classes offer better linear separability [12]. This could give rise to good prediction performance even when the input features apparently do not contain information to differentiate the target classes. Obstetricians often consider this type of classification algorithm as a black box or a mathematician’s gadget due to its being difficult to interpret [13], and so find it difficult to trust the predictions of these complex classifiers. By contrast, obstetricians are familiar with linear discriminant analysis (LDA), logistic regression (LR) and k-nearest neighbors (KNN) [12], which are simple and easy to interpret [13]. In addition, since the nonlinear transformation of the data is avoided, the definition of input EHG features used to obtain the prediction model also contributes to a better understanding of the uterine electrophysiological mechanism associated with labor. It is therefore fundamental to develop preterm labor prediction systems using simple and easily interpretable algorithms to improve the transferability of the EHG technique to clinical practice by gaining obstetricians confidence in prediction model outcomes [14]. Secondly, due to using reduced sample sizes, many previous studies have used cross-validation methods to design and validate the classifiers, without determining the real generalization capacity for incoming data ‘never’ seen by the classifiers [7].

The aim of this work was therefore to develop easily interpreted prediction systems based on EHG features for forecasting preterm labor in women at regular check-ups and to determine its generalization capacity for incoming data ‘never’ seen by the classifiers, to facilitate the transfer of this technique to clinical practice. We also attempted to identify those EHG features that presented relevant and complementary information on labor prediction.

## 2. Materials and Methods

### 2.1. Database

**Figure 1:**
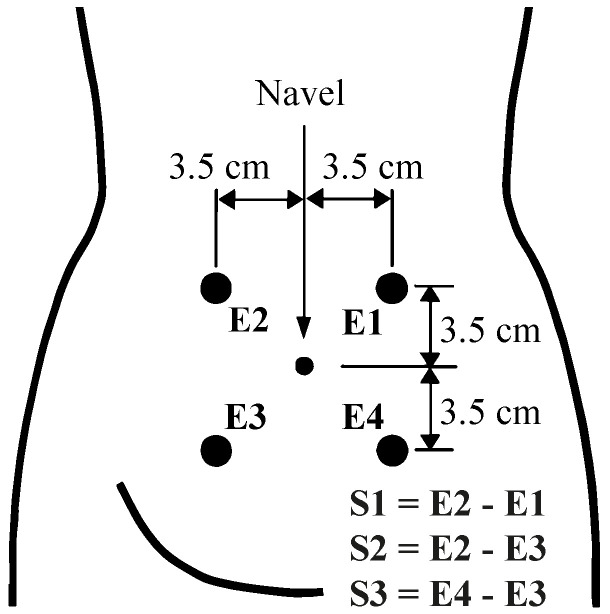
Recording protocol of EHG signals. Modified from [15].

Two public EHG databases available in Physionet were used for the study: The “Term-Preterm EHG Database” (TPEHG DB) [14] and the “The Term-Preterm EHG Dataset with tocogram” (TPEHGT DS) [15]. Both databases were obtained by the Department of Obstetrics and Gynecology at the Ljubljana University Medical Center. To agree with the data available for each patient, only EHG recordings from both databases were used to predict preterm labor, i.e. the tocogram signals included in THEHGT DS were ignored. They comprised a total of 326 EHG signals from 275 term labor (labor > 37 weeks) and 51 preterm labor were recorded during routine checkups of pregnant women between 22 and 37 weeks of gestation. The protocol used to obtain the EHG recordings consisted of placing four disposable electrodes on the woman’s abdomen at an interelectrode distance of 7 cm. Three bipolar channels (S1, S2 and S3) were obtained from the monopolar EHG recordings, as shown in Figure 1. Each signal was digitized at 20 samples per second per channel with a 16-bit resolution over a range of ±2.5 millivolts [14]. A demographic description of both databases was provided in Table 1.

### 2.2. EHG Signal Characterization

Physiologically, various types of uterine contractions such as Alvarez waves, Braxton Hicks contractions, preterm contractions, and with less frequency the so-called “long duration low-frequency band waves” [16] may be present in EHG recordings acquired from pregnant woman in the third trimester of gestation not close to delivery. The amplitude of the EHG bursts associated with these uterine contractions is expected to be very low, giving rise to subtle changes from basal activity. It is therefore very difficult to accurately identify the onset and offset of uterine contractions in these recordings and this could generate some uncertainty in the results derived from them. Previous results have revealed that whole window analysis can also be used for characterizing EHG signals [8,14,17] and that it even outperforms EHG-burst analysis for predicting imminent labor in women with threatened preterm labor [18]. Whole window analysis has the additional advantage of not requiring the uterine contractions to be identified in the EHG recordings, and only non-physiological segments should be excluded (such as artifacted segments and those with respiratory interference), thus facilitating its implementation in real time. In this work we therefore performed whole EHG window analysis to characterize the EHG signals rather than EHG-burst analysis. Two experts identified physiological segments in the EHG recordings by a double-blind process. The EHG characteristics of the recordings were analyzed in 120 s windows with a 50% overlap, the window length being a tradeoff between computational cost and preserving the representative segment of the recordings [18]. We then computed the median value of all the analyzed windows in the recording to obtain a single representative value of each EHG parameter per recording.

A total of 66 temporal, spectral and non-linear parameters were worked out per recording channel and session, see Table 2. Firstly, the EHG signal is known to contain Fast Wave Low (FWL) and Fast Wave High (FWH) components, which are associated with signal propagability and cell excitability, respectively, their energy being distributed in 0.2–0.34 Hz and 0.34–4 Hz, respectively [6]. Due to the relatively lower signal-to-noise interference above 1 Hz [6,14], we considered both 0.34–4 Hz and 0.34–1 Hz to characterize the FWH component. Therefore, we calculated the peak-to-peak amplitude (App) to describe the signal amplitude of different EHG components in four bandwidths: whole EHG bandwidth 0.1–4 Hz; FWH bandwidth 0.34–4 Hz; 0.2–0.34 Hz and 0.34–1 Hz, in which the energy of the FWL and FWH components is mainly distributed respectively. Due to the increasing formation of gap junctions as pregnancy progresses, signal amplitude, which is associated with the number of uterine cells involved in one contraction, was shown to increase as labor approaches [6].

As labor gets nearer, the EHG signal spectral content shifted to higher frequencies, suggesting increased cell excitability [6]. Different spectral parameters have been proposed to quantify the signal spectral content distribution: mean frequency (MeanF), dominant frequency (DF) computed in the range 0.2–1 Hz and in 0.34–1 Hz, power spectrum deciles (D1, . . . , D9), normalized energy (NormEn) (0.2–0.34 Hz, 0.34–0.6 Hz 0.6–1 Hz) [19], high-to-low frequency energy ratio (H/L ratio) and spectral moment ratio (SpMR), as in [15]. We also included Teager energy, which contains information not only on signal amplitude, but also on the frequency content [8]. Due to the increased cell excitability, different spectral parameters are expected to increase as pregnancy progresses, however there is no agreement in the literature about the spectral parameters that can best characterize the EHG signal, and above all those that provide information to complement temporal and non-linear parameters.

Due to the non-linear nature of the biological process dynamics, non-linear parameters have been widely used to characterize EHG signals. A previous study showed that the bandwidth in which the non-linear parameters are computed is a key factor in obtaining a robust and physically interpretable indicator for characterizing EHG signals [14,17]. We therefore computed several non-linear parameters in the same four bandwidths as App to determine whether there was any redundant or complementary information between non-linear parameters computed in different bandwidths and to other linear features. SampEn, FuzEn and SpEn were used to measure time series regularity and predictability in both the time and frequency domains [8,14]. A lower value of entropy metrics is associated with more self-similarity in the regular and predictable time series. We also computed the Lempel-Ziv index (binary (LZBin) and multistate n = 6 (LZMulti)), which evaluates time series complexity by measuring the ‘diversity’ of the patterns embedded in a time series [8,18]. Time reversibility (TimeRev) estimates the similarity in forward (natural) and reverse time and can be considered as a measurement of the degree of signal nonlinearity [8]. Uterine myoelectric activity has also been shown to possess fractal properties and is another way of measuring signal self-similarity [20,21]. We also computed Katz’s fractal dimension (KFD) [22] since it is less sensitive to noise than Higuchi’s method [23]. It was defined as the ratio between the curve length, which corresponds the sum of the Euclidean distances between successive points of the time series, and the maximum distance between the first point and any sample of the time series [22].

Since the ‘present’ EHG signal amplitude may significantly influence the ‘following’ values, we represented the Poincaré plot of consecutive EHG signal amplitudes (EHG[n] vs. EHG[n−1]) to estimate the short (SD1) and long-term (SD2) variation of the dispersion along the minor and major axes of the ellipse, respectively [10]. We then obtained the SDRR, defined as square root of the variance of the whole time series $\sqrt{(\mathrm{SD}1^{2}+\mathrm{SD}2^{2})/2}$ and SD1/SD2 ratio, which measures signal randomness. This latter has been shown to decrease significantly in women with threatened preterm labor who delivered in less than 7 days, in comparison to those who delivered in more than 7 days [8]. Table 2 summarizes the EHG parameters and obstetric data used to design the preterm labor prediction model (3 channels · 66 EHG parameters per channel + 5 obstetric data = 203 features).

### 2.3. Classifier Design and Evaluation

Since the preterm birth rate is about 12% in women who have regular check-ups, this means that the two target classes are highly imbalanced. It is well known that the conventional classification algorithms are often biased towards the majority class for imbalanced data, obtaining a higher misclassification rate for the minority class instances [24]. In this case, low sensitivity can be expected for true preterm labor. In this work, we used the synthetic minority oversampling technique (SMOTE, k = 5) [24], which has been widely used to mitigate imbalanced class problems, to obtain balanced preterm labor and term labor data [7].

We then used the conventional holdout method (30 partitions) to design and validate the classifiers. For each partition we randomly split the whole balanced database into 3 datasets with the same proportion between the classes: training (1/3), validation (1/3) and testing (1/3) for designing, validating and testing the classifier, respectively.

As mentioned above, a total of 203 EHG features derived from the 3-channels EHG recording and obstetric data was used to design the prediction system. Since they may contain mutual and redundant information or noise, which could lead to loss of prediction performance, it is fundamental to reduce the dimension of the data. The relevance of the features for predicting preterm labor can be evaluated either individually (unidimensional approaches) or multidimensionally. Unidimensional approaches are simple and fast and therefore appealing. Nevertheless, if we only consider a unidimensional approach, the outcomes suggest eliminating those with non-significant statistical differences. The individual discrimination power must not be the only consideration since possible correlations and dependencies between the features are not considered. Many authors [12,25,26,27,28] claim that the redundant information shared between the characteristics leads to discarding some of them with a feature selection algorithm. The same occurs with noisy features that add an artifact to the classification. Although complementarity between features is critical to achieve good performance, multidimensional search techniques such as mutual information estimation may be helpful to evaluate possible correlations and dependencies between features. Nevertheless, estimating the mutual information (especially through probability density function estimations) between high-dimensional variables is a hard task in practice due to the limited number of available data points for real-world problems [29]. In this work, we measured the capability of each individual feature to identify a premature delivery with a statistical test. The Wilcoxon Rank-Sum Test was performed to compare the features’ ability to distinguish term and preterm deliveries from EHG recordings in routine check-ups. This is a non-parametric statistical hypothesis test used to compare two related samples to assess whether their population mean ranks differ (α < 0.05) [30]. Lower *p*-values mean higher discriminatory capacity between target classes for the individual feature. We performed multidimensional analysis by using a wrapper method for feature selection which has been widely used in the literature [25,27,31] and has been proven to provide better results than filter methods based on the intrinsic information embedded in the features: the genetic algorithm [31]. The initial feature set was assessed by a genetic algorithm to fit with logistic regression (LR), linear discriminant analysis (LDA) and k-nearest neighbour (KNN) classifiers. If a feature was selected by one or several classifiers, it meant that it complemented others in predicting preterm delivery. The genetic algorithm is a random search strategy based on procedures of natural evolution and is widely used for selecting the optimal feature subset to design computer-aided systems for pattern recognition in different biomedical applications [32,33]. In this work, population size (N) and genome length were fixed to the number of model features, N = 203 [26]. The crossover function implemented was the arithmetic crossover with a probability of 0.8. Typically, it was assumed between 0.6 and 1, increasing the randomness of the children generation the lower the value it takes [34]. We used mutation uniform for the mutation function, with a probability of 0.01, since the convergence to a lower minimum is better with low values (<0.1) [31,34]. The tournament function with a size of 2 and an elite count of 2 was used to select the next generation population [26]. The termination condition of the genetic algorithm was defined as a differential tolerance of $10^{-6}$ for the fitness function between 150 consecutive generation’s best fitness value.

**Figure 2:**
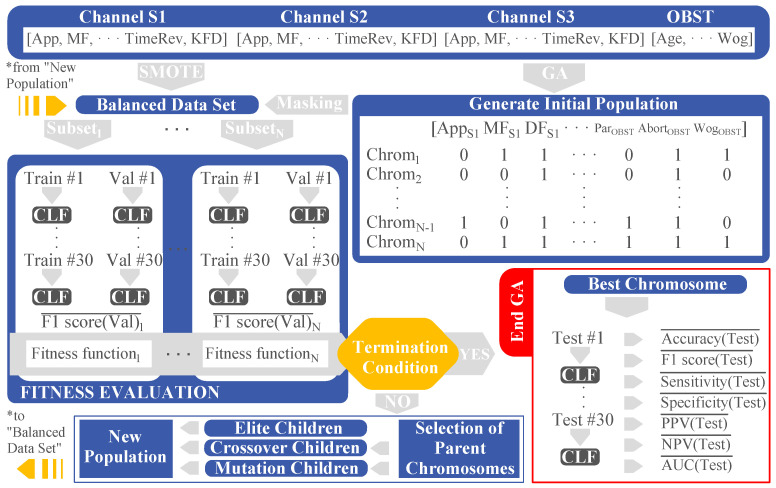
Genetic algorithm for selecting optimized feature subset to predict preterm labor based on EHG. Considering: genetic algorithm (GA), classifier (CLF), training partition (Train), validation partition (Val), testing partition (Test), chromosome (Chrom), population size (N), obstetrics features (OBST).

Figure 2 summarizes the procedure carried out to fit the model and obtain the optimized feature subset. Firstly, an initial population was randomly established, then the balanced data set was masked with the chromosomes, obtaining the different feature subsets. The mask (selected features), which corresponded to an i-chromosome, was set to the balanced data set obtaining the i-subset, with 1 ≤ i ≤ N. Subsequently, for each feature subset, the training dataset was used to fit the prediction model for each partition. The average F1-score over 30 partitions in validation subsets was then worked out to assess the model’s performance. When all the chromosomes in the the population were evaluated, a new one was created, crossing over and mutating the last population and keeping the chromosomes that outperformed the fitness function (see Equation (1)). It became an iterative procedure until the termination condition was reached, giving rise to the optimized feature subset which corresponded to the best chromosome that optimized the fitness function. Finally, we determined the model’s generalization capacity for the testing dataset which could be considered as the incoming new data ‘never’ seen by the model.

(1) $$\mathrm{Fitness}\;\mathrm{function}=\max\{\bar{F1-\mathrm{score}}\cdot \left(\mathrm{NFeat}-\mathrm{NCFeat}\right)\}$$

where $\bar{F1-\mathrm{score}}$ is the average F1-score of validation dataset for 30 partitions, NFeat is the number of features of the initial set and NCFeat is the number of features of the current subset.

For the classification methods, we compared different methods (LDA, LR and KNN) that can easily be interpreted by obstetricians. Due to the fact that the optimization cost function of the genetic algorithm is the weighted classifier performance (1) [26], different optimized EHG feature subsets may be obtained for each classification method.

To compare the different prediction model’s performance the following metrics were used: accuracy, F1-score, sensitivity, specificity, positive predictive value (PPV), negative predictive value (NPV) and area under curve (AUC) [12]. The Friedman nonparametric test was conducted to determine the statistical difference in different metrics [35]. The Nemenyi post-hoc test [35] was then used for pair-wise evaluation of the classifiers, checking the similarity of their performance.

Since individual classifiers may present a high bias or variance, giving rise to weak base models, to overcome these problems we also evaluated the utility of ensemble classifier to achieve better performance. The three previously obtained base models based on LDA, LR and KNN were used, with a commonly used majority voting strategy in the meta-level to obtain a more robust meta-classifier preterm labor predictor. We also determined the model’s generalization capacity for testing incoming data ‘unseen’ by the model for the ensemble classifier. Base and ensemble classifiers performance variability were compared by computing the coefficient of variation of the classifier metrics for both the validation and testing dataset.

## 3. Results

**Figure 3:**
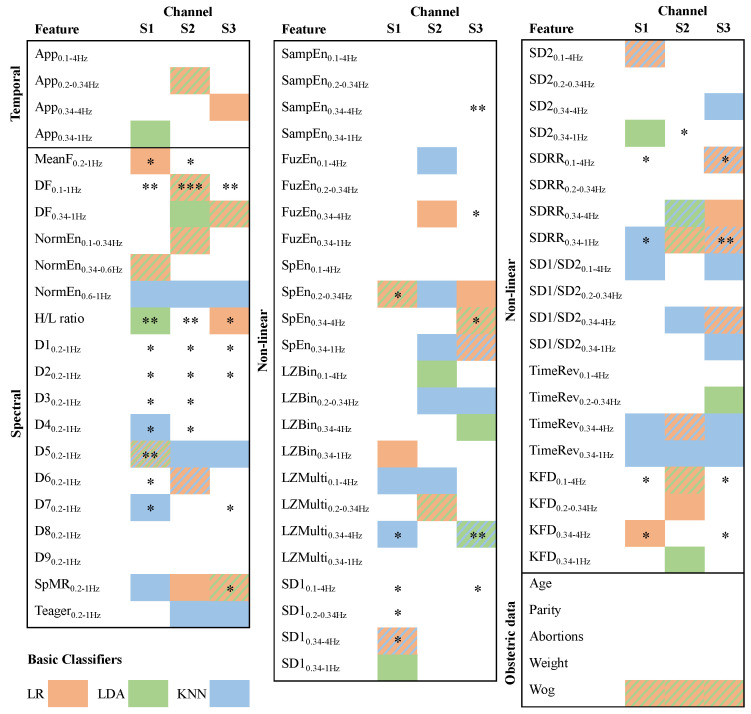
Optimized feature subset (best chromosomes) achieved using genetic algorithm for each base classifiers implemented with LR (orange), LDA (green), and KNN (blue). A single frame color indicates that a feature is part of the classifier’s best chromosome identified with that color; two and three frame colors indicate that the feature belongs to two and three classifiers’ best chromosome, identified with the corresponding colours. *, ** and *** mean a *p*-value of the Wilcoxon Rank-Sum test lower than 0.05, 0.005 and 0.0005, respectively, computed by each feature between preterm and term labor classes.

Figure 3 shows the three optimized feature subsets for predicting preterm labor using LDA, LR and KNN as classification methods. It also shows the outcomes of individual correlations of the Wilcoxon Rank-Sum test for each individual feature to discriminate between preterm and term labor classes. Firstly, we found that the EHG features computed from the 3 channels contained complementary information: 22, 25 and 23 features computed from S1, S2 and S3 respectively were included in at least one of the three optimized feature subsets. Peak-to-peak amplitude computed in the 0.2–0.34 Hz bandwidth, where the main energy of the FWL component is concentrated, seems to provide relevant information for predicting preterm labor, having been included in the “best chromosomes” of the LR and LDA classifiers. In general, there is important mutual information between different spectral EHG parameters. In this regard, deciles D1–D3, D8 and D9 were not included in the best chromosome of any of the three base classifiers. The DF in 0.1–1 Hz and 0.34–1 Hz, NormEn in 0.1–0.34 Hz and 0.34–0.6 Hz, D5, D6 and SpMR seem to provide most relevant information on cell excitability. As for the non-linear parameters, the information extracted from different bandwidths was not necessarily redundant, but rather complementary. The non-linear parameters, extracted from 0.34–4 Hz, where FWH components distributed their energy, seem to contain the most relevant information for predicting preterm labor. In contrast, the non-linear parameters computed from 0.2–0.34 Hz contain less complementary information for differentiating preterm and term records. In this respect, the parameters derived from the Poincare plot, SampEn and FuzEn, computed in this bandwidth were not included in any of the three base classifiers. In comparison to LZBin, LZMulti seems to provide more complementary information to other EHG features and therefore more present in the best chromosome of the different algorithms (see Figure 3). The entropy metrics (SampEn, FuzEn and SpEn) contained redundant information, SpEn being the most relevant one forming part of the optimum feature subset of several classifiers. Both TimeRev and KFD also offered relevant and complementary information to other features. As for the common features shared between the different prediction models, only decile 5, which is equivalent to median frequency, was common for the best chromosome of the three base classifiers. It can be seen that a subset of 11 and 8 features was also shared by LR and LDA, and by LR and KNN classifiers, respectively. As for obstetrics data, only Wog was shown to be relevant for predicting preterm labor for both LDA and LR classifiers. As for individual statistical test, a total of 43 features with statistically significant differences were obtained. Analysing the results by features selected by classifiers, a total of 29, 22 and 40 characteristics for LR, LDA and KNN were chosen respectively. Only 17 of these features that obtained a *p*-value < 0.05. 21 features were selected by at least 2 classifiers and 8 of these obtained a *p*-value lower than 0.05. Not all those features which obtained a *p*-value < 0.05 (decile 1 to 3 or sample entropy) were included in the optimized feature subset. Various features which provided individual statistical significance between target classes were used in each optimized feature subset. Likewise, the optimized feature subsets also included some features that did not obtain individual statistical significance between target classes but provided complementary information to other relevant features, for example peak to peak amplitude, Teager, SD ratio or time reversibility.

**Figure 4:**
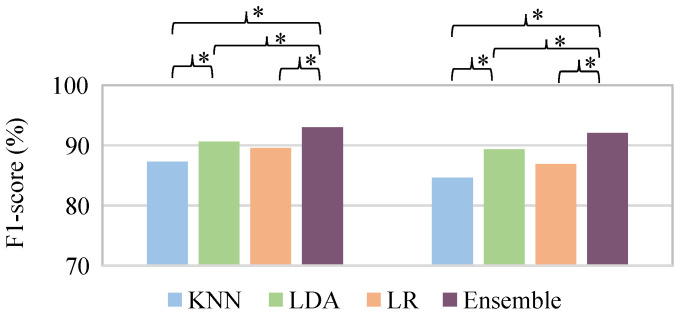
Nemenyi post-hoc test of F1-score between the different classifiers for both validation (**left**) and testing (**right**) dataset. * means significant statistical difference (*p*-value ≤ 0.05) between classifier performance.

**Figure 5:**
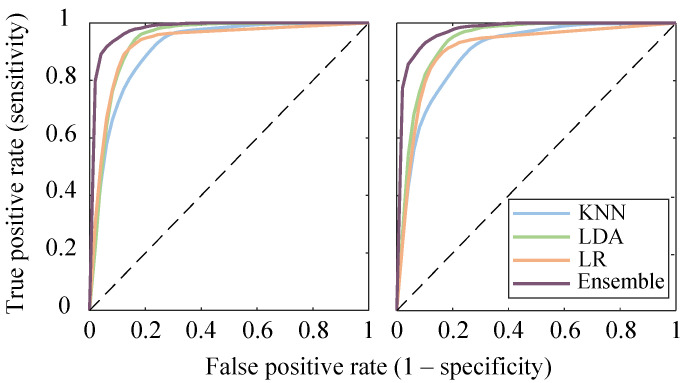
ROC curves of the different base and the ensemble classifiers using optimized feature subset for both validation (**left**) and testing dataset (**right**).

Table 3 shows the average performance for both base and ensemble classifiers for training, validation and testing dataset and Figure 4 shows the results of the Nemenyi post-hoc test of F1-score between different classifiers for both validation and testing datasets. In general, the base classifiers performance for training dataset was better than the validation dataset, as expected. The classifier metrics of the testing dataset was similar to or slightly inferior than the validation dataset. Nevertheless, regardless of the classification method, the average F1-score was over 85% and 80% for validation and testing dataset, respectively. The LDA and LR classifiers showed similar performance and obtained no significant difference. Both LDA and LR classifiers obtained better performance than KNN, although a significant difference was only achieved for the LDA classifier. The ensemble classifier obtained a significantly better performance than the different base ones, achieving an average F1-score of around 92% for both the validation and testing datasets. The receiver operating characteristic (ROC) curves of the different classifiers for validation and testing partitions are shown in Figure 5.

**Figure 6:**
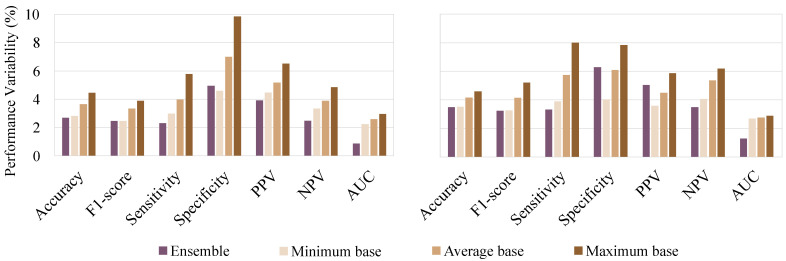
Base and ensemble classifiers performance variability for both validation (**left**) and testing (**right**) dataset. As for base classifiers, minimum, average and maximum variability were computed.

As for classifier performance variability between partitions, training data usually obtained a lower value than the validation and testing datasets with similar results. In Figure 6 are depicted the different classifier metrics’ variability for both validation and testing dataset are depicted. In general, base and ensemble classifiers presented low performance variability (<10%). Accuracy, F1-score and AUC metrics usually achieved the lowest variability, while sensitivity, specificity and PPV generally had the highest. It can also be seen that the ensemble classifier can considerably reduce the different metrics’ variability, obtaining similar or lower values than that of the minimum variability achieved by the base classifiers.

## 4. Discussion

Our aim in this work was to develop a preterm labor prediction system based on EHG using simple and easily interpretable classification algorithms in order to promote the transfer of the EHG technique to clinical practice. In this context, the features’ quality, i.e. their capability to provide useful and complementary information to others, it is critical to achieve satisfactory classification performance. Although temporal, spectral and non-linear EHG parameters have been shown to provide relevant information for predicting preterm labor [7], the redundancy and complementarity of different EHG features were still unclear. The classical dimension reduction methods, such as principal component analysis (PCA), does not guarantee the extraction of complementary information or noise reduction to optimize classifier accuracy [36].

Unlike the filter methods for feature selection that reduce the number of features using the intrinsic properties of the data, regardless of the learning algorithm to be used, we proposed to use wrapper methods, which generally lead to better classification performance [27] to determine the optimized feature subset. Of the different search strategies, we preferred to use the random search strategy, which is a tradeoff between classification performance and search complexity for moderate and/or large numbers of features [27]. In this respect, both particle swarm optimization (PSO) and the genetic algorithm could be used to optimize data information in feature space. Benalcazar et al. proposed the use of PSO and the neural network to predict labor induction success [37]. Alamedine showed that PSO generally outperformed sequential forward selection and Jeffrey divergence distance for predicting labor and pregnancy contractions when using LDA, QDA and KNN as classification methods [25]. The genetic algorithm has also been shown to obtain better performance than the filter method to predict pregnancy and labor contractions using KNN [38], and also outperformed both forward and backward selection for predicting central nervous system embryonal tumor outcomes, based on gene expression [27]. This is due to the ability of the genetic algorithm to escape local optima and discover the global optimum in even a very rugged and complex fitness function [31]. In practice, the genetic algorithm may not always lead to a theoretically perfect solution to a problem, but always delivers at least a very good solution [31]. We here used the genetic algorithm to perform feature selection as it had been shown to theoretically outperform PSO in obtaining highest number of best minimum fitness and did so faster [39]. We believe that in our context, using the PSO strategy would give rise to similar or slightly worse results.

Using the genetic algorithm for feature selection, we proved the feasibility of developing a preterm labor prediction system with high generalization capacity using simple and easy-to-interpret algorithms, achieving an F1-score of the individual base classifier of over 80% for incoming data previously unseen by the model. Since these simple classifier’s success depends mainly on the information embedded in the feature, we believe that this will help to gain obstetricians confidence of preterm labor prediction model’s outcome, bringing thus the EHG technique closer to clinical practice. The prediction model proposed here has the inherent advantage over PCA that it does not require all the parameters to be computed once the model is trained, and is therefore easier to implement on a portable device due to its lower computational cost. In this respect, the time necessary to compute the optimized feature subsets of each window was 19.03 seconds using just 1 core of an Intel Core I7 8550U laptop, which can be considerably less than the maximum time limit of 60 s (step size between analysis windows). In addition, the total time consumption required for each base classifier implemented by LR, LDA and KNN to obtain its outcome from the input features applying a majority voting strategy to generate the ensemble classifier’s outcome was 0.094 s, indicating that the prediction outcome can be obtained immediately after the recording. To characterize the EHG signal, the median value of the temporal, spectral and non-linear parameters was worked out in windows of 120 s as the representative value of the whole recording. Previous studies have shown that the 10-90th percentiles of individual EHG parameters can better discriminate between preterm and term records [17]. The prediction model’s performance when using these percentiles of EHG features (results not shown for the sake of brevity) was similar to that obtained in this work with no significant differences.

We also reported for the first time three EHG feature subsets (best chromosomes) that contain the maximum complementary information, thus optimizing the prediction model’s performance. Our results revealed the redundancy between different spectral parameters and also the complementarity between temporal, spectral and non-linear parameters. This result is understandable, since these represent the different phenomena involved in uterine contraction efficiency: intensity, excitability and non-linear dynamic character [6]. As for non-linear parameters, Fele-Žorž et al. showed that SampEn computed in FWH bandwidth can discriminate preterm and term records [14]. Lemancewicz et al. found that Lempel–Ziv and approximate entropy computed at 0.24–4 Hz was significantly higher for women with threatened preterm labor who delivered in less than seven days than in those who delivered in more than seven days [9]. On the other hand, Lempel-Ziv computed at FWH bandwidth in women with threatened preterm labor decreased as labor approached [18]. Mas-Cabo et al. compared different non-linear parameters computed from the whole bandwidth (0.1–4 Hz) and fast wave high bandwidth (0.34–4 Hz) and concluded that the signal bandwidth in which non-linear parameters are computed may be a key factor in obtaining a robust and physically interpretable indicator for characterizing EHG signals [18]. In this work, we found that the non-linear parameters computed in different bandwidths provided complementary information. In addition, SampEn, FuzEn and Lempel-Ziv which represent the signal predictability and complexity in time domain, contained redundant information between each other. SpEn, which measures the flatness of the spectrum, seemed to provide additional information on the signal. Of the obstetric data, only gestational age was found to be relevant for predicting preterm labor, possibly due to the values of EHG features being intrinsically modified as gestational age increases [14]. However, maternal age, parity and abortions were irrelevant for the algorithm, although these latter have been associated with preterm labor risk factors [40]. We believe that other obstetric data, such as fetal fibronectin and cervical length may provide complementary information to EHG and improve preterm labor prediction performance.

The results revealed the complementarity of EHG features extracted from different channels, highlighting the utility of multichannel recording for preterm labor prediction. Firstly, a similar number of features from the three channels were included in the optimized feature subsets (Best chromosomes). Using the same method (Genetic algorithm + LDA, LR or KNN classifier), we also attempted to develop a preterm labor prediction system using the information extracted from individual channels and computing a mean efficiency index, which has been shown to be a more robust indicator of uterine electrical activity efficiency from multichannel recordings [41]. We found that the model performance based on individual channels was much inferior (<70%) to that obtained for multichannel recording, suggesting that multichannel recording may provide a more reliable electrophysiological state of the whole uterus [41]. Unlike the prediction system based on a neural network developed in a previous work, we obtained a better performance using all the EHG features extracted from the three individual channels (S1+S2+S3) than the mean efficiency index [41]. We believe this discrepancy may be associated with the greater degree of freedom in combining information from a multichannel recording and the genetic algorithm’s capacity to optimize the information in feature space. Our results for base classifiers also outperformed those obtained by Fergus, who used PCA to reduce the data dimension of EHG features extracted from channel 3 in the 0.34–1 Hz bandwidth. An AUC of 66%, 86% and 84% was obtained from this latter to validate the dataset using LDA, LR and KNN classifiers, respectively [42]. Our results are also comparable with those obtained using a neural network [7] and PCA for data dimension reduction (AUC = 88.2%). In other words, the information optimization in the feature space eliminated the need to use complex classification methods. In comparison with other studies in the literature [43,44], our prediction model’s performance may be slightly lower, which could be due to the different method used to validate it. In this respect, the use of cross-validation and hold-out validation without preserving a testing dataset previously unseen by the model may overestimate the model performance [12].

We also evaluated the performance improvement in a ensemble classifier over base classifiers in predicting preterm labor. Ensemble methods combining the output of individual weak classifiers have successfully produced accurate predictions for many complex classification tasks [12]. The success of these methods is attributed to their ability to consolidate accurate predictions and correct errors across many diverse base classifiers [45,46]. Successful ensemble methods make a balance between the ensemble’s diversity and accuracy [47]. In this work we showed that a simple ensemble classifier aggregation whose meta-level only consisted of a majority voting strategy can further improve classification performance, obtain higher average metrics and reduce the different metric variabilities between partitions. In this respect, the use of other meta-learning algorithms may further improve classification performance. Again, it is preferred to use simple and easy-to-interpret algorithms to develop the meta-classifier. Future work will compare different meta-learning algorithms in terms of improved classification performance and to further validate the utility of these methods for predicting imminent labor and/or preterm labor in women with threatened preterm labor undergoing tocolytic treatment. Regardless of the improvement in performance achieved by using ensemble classifiers, obstetricians may find it significantly more difficult to interpret these algorithms, and this should be taken into account when transferring the EHG technique to clinical practice.

In spite of its promising results, the present study is not exempt from limitations. Firstly, the size of the samples of women who took regular check-ups and delivered at term or prematurely was highly imbalanced. We used the commonly-used SMOTE oversampling technique to minimize this problem [24]. Specific imbalanced data learning algorithms, such as weighted classifiers or boosting ensemble learning, could help to achieve more reliable preterm labor prediction systems. Secondly, due to the limited sample size, a larger database is still needed to further validate the performance of preterm labor prediction systems before transferring them to clinical practice. Despite these limitations, we believe that this work constitutes a significant step towards putting the EHG technique into clinical practice.

## 5. Conclusions

By optimizing feature subspace with genetic algorithms, we showed the feasibility of developing a preterm labor prediction system with a high generalization capacity using simple and easy-to-interpret algorithms such as LDA, LR and KNN, obtaining an average F1-score of 89.34 ± 3.5%, 86.87 ± 4.53% and 84.63 ± 2.76%, respectively, for the testing dataset. We found that temporal, spectral and non-linear EHG parameters computed in different bandwidths provide complementary information for predicting preterm labor. In addition, we further proved that the information extracted from multichannel recordings was also complementary among channels. A simple aggregation ensemble classifier can obtain more reliable preterm labor prediction systems than individual weak base classifiers, achieving higher average metrics and lower variability between partitions. The average F1-score of the ensemble classifier was about 92.04 ± 2.97% for an incoming new dataset previously unseen by the model, which was significantly higher than that of commonly used obstetric techniques.

The optimized feature subset requires a few further features to be computed, which, together with use of easily interpreted classifier algorithms, would contribute to implementing preterm labor prediction systems in real-time and improve clinical staff’s acceptance of the EHG technique, thus promoting its transferability to clinical practice.

## Figures and Tables

**Table 1:** Demographic description of the both databases (TPEHG DB and TPEHGT DS).

Group	N	Maternal Age (Years)	Parity	Abortions	Maternal Weight (kg)	Wog * (Weeks)	Birth (Weeks)
Term	275	29.33 ± 4.34	0.40 ± 0.74	0.23 ± 0.61	68.55 ± 10.55	26.95 ± 4.19	39.21 ± 1.12
Preterm	51	29.08 ± 5.26	0.41 ± 0.64	0.26 ± 0.60	66.82 ± 11.24	27.59 ± 3.72	33.92 ± 2.21

* Gestational age at the moment of the recording (weeks).

**Table 2:** Input features for predicting preterm labor that including both temporal, spectral and non-Linear EHG parameters and obstetric data.

EHG Temporal Parameters	EHG Spectral Parameters	EHG Non-Linear Parameters	Obstetric data
		SampEn	
		FuzEn	
	MeanF	SpEn	Maternal age
	DF	LZBin	Parity
App	NormEn	LZMulti (n = 6)	Abortions
	H/L Ratio	SD1	Weight
	[D1, . . . , D9]	SD2	Week of gestation (Wog)
	SpMR	SDRR	
	Teager Energy	SD1/SD2	
		TimeRev	
		KFD	

**Table 3:** Average performance of 30 partitions for base classifiers (LDA, LR and KNN) and for the classifiers’ ensemble.

		KNN	LDA	LR	Ensemble
>Accuracy (%)	Train	92.86 ± 2.41	96.01 ± 1.50	100 ± 0.00	99.62 ± 0.59
	Validation	85.82 ± 3.82	90.03 ± 2.53	89.78 ± 3.29	92.61 ± 2.48
	Test	82.92 ± 2.90	88.77 ± 3.89	87.42 ± 4.00	91.64 ± 3.20
>F1-score (%)	Train	93.26 ± 2.20	96.14 ± 1.43	100.00 ± 0.00	99.63 ± 0.58
	Validation	87.26 ± 3.21	90.61 ± 2.23	89.55 ± 3.49	92.97 ± 2.27
	Test	84.63 ± 2.76	89.34 ± 3.50	86.87 ± 4.53	92.04 ± 2.97
>Sensitivity (%)	Train	98.43 ± 1.86	98.99 ± 1.29	100.00 ± 0.00	99.87 ± 0.48
	Validation	96.48 ± 2.88	95.79 ± 3.04	87.86 ± 5.07	97.36 ± 2.25
	Test	94.21 ± 5.00	93.58 ± 3.63	84.03 ± 6.73	96.23 ± 3.17
>Specificity (%)	Train	87.30 ± 4.05	93.02 ± 2.81	100.00 ± 0.00	99.37 ± 1.14
	Validation	75.16 ± 7.40	84.28 ± 5.48	91.70 ± 4.22	87.86 ± 4.34
	Test	71.64 ± 4.58	83.96 ± 6.59	90.82 ± 3.67	87.04 ± 5.47
>PPV (%)	Train	88.67 ± 3.28	93.48 ± 2.49	100.00 ± 0.00	99.39 ± 1.11
	Validation	79.83 ± 5.19	86.12 ± 3.95	91.51 ± 4.08	89.04±3.49
	Test	76.95 ± 2.76	85.63 ± 5.02	90.2 ± 3.58	88.33 ± 4.44
>NPV (%)	Train	98.25 ± 2.09	98.95 ± 1.33	100.00 ± 0.00	99.88 ± 0.47
	Validation	95.64 ± 3.32	95.41 ± 3.18	88.50 ± 4.28	97.13 ± 2.40
	Test	92.92 ± 5.42	92.99 ± 3.78	85.33 ± 5.28	95.94 ± 3.35
>AUC (%)	Train	98.49 ± 0.83	99.30 ± 0.56	100.00 ± 0.00	100.00 ± 0.00
	Validation	92.16 ± 2.37	94.72 ± 2.10	93.03 ± 2.74	98.63 ± 0.85
	Test	90.20 ± 2.41	94.72 ± 2.54	91.44 ± 2.63	98.13 ± 1.26

## Data Availability

The data used to support the findings of this study are included within the article.
